# Territorial Polemics: A Response to Roberts

**DOI:** 10.1007/s10764-012-9605-4

**Published:** 2012-05-24

**Authors:** Yvan Lledo-Ferrer, Fernando Peláez, Eckhard W. Heymann

**Affiliations:** 1 Abteilung Verhaltensökologie & Soziobiologie, Deutsches Primatenzentrum, 37077 Göttingen, Germany; 2 CAPP (Centro de Administração e Políticas Públicas), Instituto Superior de Ciências Sociais e Políticas, Universidade Técnica de Lisboa, 1300-663 Lisboa, Portugal; 3 Área de Psicobiología, Facultad de Psicología, Universidad Autónoma de Madrid, 28049 Madrid, Spain

**Keywords:** Communication, Costs and benefits, Scent-marking, Tamarins

## Abstract

Our recent paper Lledo-Ferrer *et al*. (International Journal of Primatology 32: 974–991, 2011) questioned the classic view of territoriality and chemical communication in wild callitrichids, saddleback tamarins (*Saguinus fuscicollis*). We suggested that rather than defending a territory or resources, chemical communication was more likely to be a way of exchanging reproductive information between groups. Roberts (International Journal of Primatology 33, 2012). challenged this interpretation, considering that the results could more parsimoniously be interpreted as fulfilling a resource defense strategy. This response is intended to clarify some aspects of the debate and to suggest how further research could shed new light on the present polemics.

tum ex composito orta uis, signoque dato iuuentus Romana ad rapiendas uirgines discurrit.

Titus Livius, Ab urbe condita, I,9

An abundant literature on chemical communication in mammals has demonstrated a close link between scent-marking and territoriality (Brown and Macdonald 1985; Gosling 1982). Animals usually react aggressively against strangers and scent-mark to advertise potential costs of fighting (Gosling 1990; Gosling and Roberts 2001). We had suspected for some time that the picture in tamarins (*Saguinus*, Callitrichinae) might be more complicated (Heymann 2000). In a recent field study with saddleback tamarins, (*Saguinus fuscicollis*), we suggested a new interpretation of scent-marking and territorial behavior (Lledo-Ferrer *et al*. 2011). Our data suggested that, rather than defending a territory in the classic sense —its geographic location, its feeding resources— scent-marking might be a way of exchanging reproductive and social information between groups.

Roberts (2012) disagreed with this interpretation and defended a more classic view. Here, we briefly make explicit some misunderstandings, and develop what we think are the main contentious points in discussion and the key questions to be answered in future studies.

## Costs and Benefits

Through scent-matching, an intruder is able to evaluate costs and benefits of intrusion, and adapt its behavior accordingly (Gosling 1982; Gosling and Roberts 2001). Ideally, these costs and benefits should be quantified and compared reliably. In our study, we quantified potential costs by calculating a theoretical encounter rate (Waser and Wiley 1979). If groups were moving independently, we would have found an observed rate equal to the theoretically predicted rate. If there were some kind of encounter avoidance mechanism —and scent marking is a good candidate (Moorcroft and Lewis 2006)— the observed rate should be lower than the predicted rate. We found exactly the contrary (Fig. 3, p. 983). Clearly, groups are not avoiding each other. If scent-marking is supposed to allow an assessment of neighbors without incurring the cost of fights, the scent-marking strategy of tamarins is not effective.

Roberts is nevertheless right that “there is no reason to expect that scent marks should necessarily reduce the rate of intergroup encounters” (2012), because vocal communication in tamarins probably plays a much bigger role in intergroup spacing than olfactory communication. Long-calls allow groups to locate each other, and intergroup encounters are usually preceded by such calls (Garber *et al*. 1993; Lazaro-Perea 2001). But this, in turn, argues against a territorial function of scents. Indeed, why should animals mark at all?

Meeting neighbors allows comparison of the scent and the putative signaler. This makes sense when opponents seldom meet each other. Our tamarin groups encounter each other on average every 2–3 d, group composition is stable, and so they have ample opportunities for this comparison. Why then should they tolerate the costs of meeting at such a high rate?

## Resource Defense

Even if often peaceful, intergroup encounters are characterized by frenetic activity. It seems unlikely that the additional energy gained by exclusive access to contested resources outweighs the energy expenditure during the contest. Resources in overlap areas could just be consumed when the neighbors are away. Roberts seems to disregard the observation that scents of own-group members overmarked more at feeding trees in overlap areas (p. 982 and 985–986). This is counterintuitive because the probability of the resulting mark being perceived should be lower than for two separate marks.

When resources are immediately threatened by the presence of the neighboring group, 40 % of the encounters are still peaceful (p. 983), and we have seen groups feeding on the same tree. We and others (de la Torre *et al*. 1995) have also seen tamarin “supergroups” in which two groups almost mix and move in parallel through the forest for up to 90 min. This does not seem an appropriate behavior for resource defenders. Roberts is right in highlighting that food sharing did not occur in 87 % of the encounters, but the 13 % of encounters in which resource sharing occurs still need an explanation.

Food availability does not seem to be an issue. However, to rule out this possibility, rates of intergroup encounters and of scent-marking should be compared between rich and lean seasons. If related to territoriality, we would expect a more intense resource marking strategy, and an increased frequency of territorial contests in lean seasons. It would then be worth expanding territories at the expense of neighbors. However, we found no seasonal variation in scent-marking (Lledo-Ferrer *unpubl. data*).

## Neighbors and Intruders

As Roberts points out, the different reaction of floaters and neighbors when detected is striking. It does not seem to be a question of group size. Our group 1 regularly met group 2, despite a ratio of 1:2 group members. However, we have seen two floaters fleeing when detected by the same three individuals of group 1, despite a 2:3 ratio.

If scent-matching and territorial defense were the main function of olfactory communication, then both the floater and the group should be most interested in including this new odor in their neighborhood database. However, such floaters are seldom seen and, when detected, they are immediately chased and and flee desperately, without scent-marking or even perceiving the scent from the residents.

We do not know the detection distance for scent marks and for body odors. This information would be critical for evaluating the potential for scent-matching. We do, however, assume that detection distance for scent marks is relatively low, which may be the reason why so few scent marks receive a response at all, and why such responses are more common in *Saguinus fuscicollis* than in *S. mystax*, where there are usually no other individuals close to the scent-marking individual (Heymann 2001). Further, part of the chemical information contained in scent marks seems to require close contact with the mark to elicit a full response (Belcher *et al*. 1988). Given that intruders flee at a distance and no contact between territory owners occurs, the opportunity for scent matching is likely to be very small.

Moreover, in contrast to ground mammals, the small arboreal tamarins use space in a radically different way. Whereas ground mammals move in a 2D world, tamarins move in 3D. An antelope chewing a branch and depositing saliva as scent can be sure that any other antelope passing there will find it exactly at nose height. This does not mean that all scent marks are detected, but transmission is much more effective in a 2D than in a 3D environment. Moreover, given their low body mass, tamarins have more branch paths to choose than larger primates, making their environment much more complex. To overcome such drawbacks, tamarins should mark as close to potential receivers as possible, i.e., during intergroup encounters. Roberts is right that scent-marking during intergroup encounters is enhanced, compared to 24 h later. However, this result cannot be interpreted alone. On the next day the group could be anywhere in its territory, including the exclusive area. Scent-marking frequency there is lower than in the overlap area when no neighbors are present, and thus the overall frequency is inflated by this covariate. It is thus more informative to look at the other comparison, in which the group uses an overlap area where intergroup encounters have taken place in the past, but there are currently no neighbors present. In this comparison, we found no significant difference in scent-marking frequency (Fig. 4, p. 984). Moreover, the previously deposited scents received less or no attention (p. 983–984) during the encounter.

Perhaps part of Roberts’ misinterpretation lies in the circularity of his argument. Because the sometimes aggressive confrontations with neighbors are labeled territorial defense, scent-marking is assumed to be territorial as well. But what if such confrontations were not about defense?

## Looking for Partners or Defending Resources?

With only one female successfully reproducing per group, and a high degree of relatedness within groups (Huck *et al*. 2005), intersexual competition can be extreme among callitrichines (Culot *et al*. 2011). Reproductive success is more likely to be limited by access to mates (for males) and to helpers (for breeding females) than by access to food resources. Thus, our overall interpretation is that what is at stake is probably not the takeover of the territory or its resources, but the reproductive dominance in the group. It would thus be very interesting to study whether group composition determines reactions toward neighbors and intruders. So far, the literature has considered the whole group as signaling and being involved in territorial behavior. However, different classes of individuals might have different interests and thus adopt different strategies toward neighbors. Whereas an unrelated neighboring female represents a potential partner for resident males, but a major threat for the breeding resident female, an unrelated male can be a valuable help in infant carrying for the same female, but a competitor for resident males. Moreover, nonbreeding animals should evaluate their reproductive chances within and outside the group, according to their age and relatedness to the breeding pair, and react accordingly toward neighbors. Breeding pairs, however, might be interested in obtaining extra helpers if group size is small. Lazaro-Perea (2001) found such a pattern in wild *Callithrix jacchus*, whereby subordinate individuals scent-marked more during intergroup encounters. In our study, small sample sizes do not allow us to study scent-marking behavior on the individual level.

We and others (Digby 1999; Heymann 2001) have seen extragroup copulations during intergroup encounters, and extragroup paternities do occur in moustached tamarins (Huck *et al*. 2005). As scent marks convey useful information concerning not only sex and individual identity (Epple *et al*. 1993), but also health and individual quality (Zala *et al*. 2004) and heterozygosity (Charpentier *et al*. 2008), it is not revolutionary to think that tamarins might be using this information to look for potential mates. The study of scent-marking behavior combined with kinship and relatedness data under field conditions might shed new light on scent-marking behavior. Unfortunately, this was not possible in our field study. To investigate these issues in captivity, researchers could manipulate the degree of relatedness between neighbors and the relative value of cages that might be alternatively used by different groups, or present the scent from neighbors at the core of the cage-territory (Suarez 2012).

## Conclusion

As Roberts accurately points out, our original data fit the territorial hypothesis (a border marking strategy, enhanced marking close to feeding resources and in areas where intergroup encounters are likely). However, without looking for the real benefits of the scent-marking behavior, this conclusion is superficial. We could not determine what the benefits could be, beyond theoretical cost reductions. Moreover, encounters occur on a regular basis, and scent-marking is not enhanced during these encounters. This emphasis on costs and benefits is lacking in most studies of chemical communication in the wild, and seems to be neglected by Roberts.

What if the benefit is *simply* exchanging reproductive information with neighbors? This exchange is done only during regular intergroup encounters, and not during encounters with floaters. It might be worthwhile for tamarins to engage in a fight, if this leads to a new potential partner. This is not radically different from the mythological rape of the Sabine women described by Titus Livius.

Each communication channel has its own specificities and constraints (Endler 1993). Locating a neighboring group is much more effective through auditory cues, whereas reproductive information is better codified in scents (Epple *et al*. 1993). Moreover, this reproductive information can be left behind for neighbors without own group members being aware of it, which is not the case in the auditory channel. Overmarking could be a costly way to hide this information (Johnston *et al*. 1994; Lledo-Ferrer *et al*. 2010).

Roberts is correct in pointing out that some critical data are still lacking. Given the complexity of chemical signals, it is possible that scent-marking fulfills several functions at the same time, or that each individual acts according to its own reproductive agenda. Our original paper was entitled “The *equivocal* relationship between scent-marking and territoriality.” It did not mean that a territorial function was discarded, but just that it might not be the principal function. In any case, the relationship between scent-marking and territoriality deserves closer inquiry. We hope that these territorial polemics will attract the attention of researchers and enable more careful field studies and experiments.
